# Enhancing the Simulation Speed of Sensor Network Applications by Asynchronization of Interrupt Service Routines

**DOI:** 10.3390/s130811128

**Published:** 2013-08-21

**Authors:** Hyunwoo Joe, Duk-Kyun Woo, Hyungshin Kim

**Affiliations:** 1 Department of Computer Science and Engineering, Chungnam National University, 99 Daehak-ro, Yuseoung-gu, Daejeon 305-764, Korea; E-Mail: jhwzero@cnu.ac.kr; 2 Embedded Software Research Division, Electronics and Telecommunications Research Institute (ETRI), 218 Gajeng-ro, Yuseoung-gu, Daejeon 305-700, Korea; E-Mail: dkwu@etri.re.kr

**Keywords:** networked sensors, sensor networks, optimistic simulation, synchronization, asynchronous simulation, simulation speedup, interrupt service routine

## Abstract

Sensor network simulations require high fidelity and timing accuracy to be used as an implementation and evaluation tool. The cycle-accurate and instruction-level simulator is the known solution for these purposes. However, this type of simulation incurs a high computation cost since it has to model not only the instruction level behavior but also the synchronization between multiple sensors for their causality. This paper presents a novel technique that exploits asynchronous simulations of interrupt service routines (ISR). We can avoid the synchronization overheads when the interrupt service routines are simulated without preemption. If the causality errors occur, we devise a rollback procedure to restore the original synchronized simulation. This concept can be extended to any instruction-level sensor network simulator. Evaluation results show our method can enhance the simulation speed up to 52% in the case of our experiments. For applications with longer interrupt service routines and smaller number of preemptions, the speedup becomes greater. In addition, our simulator is 2 to 11 times faster than the well-known sensor network simulator.

## 1. Introduction

Since scalable networked sensors are deployed over wide geographical areas, once they are installed, it is difficult to maintain them. Wireless sensor network simulators are very attractive and useful tools to the developers. They allow users to simulate their software under various operating conditions such as radio interference, geographical effects and functional behaviors.

Network simulators such as NS-2 [1], SensorSim [2], GloMosim [3] and QualNet [4] are used in classical sensor network simulations. They validate modeled communication protocols, but they cannot debug and verify the source codes of sensor network applications.

High fidelity of the models within simulators is needed to provide more accurate and realistic simulation results. Cycle-accurate and instruction-level simulators have been proposed for this purpose. However, these simulators have difficulties in achieving scalability as the number of the sensors increases.

In particular, ATEMU [5], the first instruction-level sensor network simulator, uses the cycle-by-cycle synchronization strategy. It simulates every event by a cycle-by-cycle strategy. Each sensor node is automatically synchronized with each other in a cycle. The cycle-by-cycle strategy can make synchronization for free but it has poor scalability. Even if some sensor nodes are asleep, all sensor nodes should be simulated in every clock cycle. On the other hand, NQEM [6] and Avrora [7] reduce the number of simulation events using discrete-event simulation. Sensor network applications generally have low duty cycles, so that simulators can skip many cycles with the method while sensor nodes are sleeping.

Another major cause of slow simulation speed is the synchronization overhead. In discrete-event simulation, each event carries a timestamp which determines the simulation time at which it is scheduled to occur. All events are processed in timestamp order to ensure causality. Virtual sensor nodes should also synchronize with each other to avoid the causality errors. Conservative [8] or optimistic approach [9] are general solutions to manage synchronization. Conservative approach demands sensor nodes to coordinate to guarantee that no causality errors can occur. In contrast, optimistic simulation enforces no explicit synchronizations. However, if a causality error is detected, a rollback procedure undoes the simulated result to the time. Every conventional sensor network simulator uses various conservative approaches to maintain the causality, even though they generate synchronization overheads. For example, NQEM manages a shared queue, and Avrora applies the lock-step technique between virtual sensor nodes.

PolarLite [10] and SnapSim [11] are extensions of Avrora to reduce the number of synchronization points because if synchronizations between sensor nodes can be avoided during simulation, the scalability can be improved. They focus on the interval for synchronization by network transmission protocols. However, sensor nodes in actual applications are usually in the sleep state, so sensor nodes do not communicate each other frequently. When sensing data is acquired or transferred, they are awakened by the events. The mentioned research did not consider these characteristics. Moreover, the solutions are dependent on the sensor network protocols and communication layers.

In this paper, we propose a novel technique for enhancing the speed of the sensor network simulations. The technique is based on the optimistic simulation approach in ISRs instead of the conservative approach. The asynchronous simulation means the sensor nodes are independently simulated regardless of other sensor nodes' status. The technique within application codes can greatly reduce the synchronization events. If there are no interrupts and any exception over a period of execution, the program execution sequence of applications is prefixed. However, interrupts can occur frequently since applications of networked sensors follow event-driven programming practices. Sensor nodes stay most of the time in the sleep mode and they are awakened when events are triggered by external interrupts such as a radio controller, sensors and actuators. Hence, the simulation of ISRs costs computation time for a large part of the code simulations. This is the main reason and motivation why the target for improvement is the interrupt service routine.

If interrupts are not preempted by other interrupts, they can be simulated asynchronously during the period of ISRs. We observed in experiments that there are only a few preemptions during ISRs in sensor node operations. For the cases in which preemptions occur afterwards during the ISR, the simulator uses an efficient rollback mechanism to restore the original synchronized simulation to maintain timing causality. The proposed method can be implemented on any instruction-level simulator by avoiding the synchronizations during simulation of ISRs. Therefore, this method can be the effective general approach for improving simulation speed. The speedup is determined by the length of the ISR and number of invoked interrupts because our technique shows better performance when the ISR is long. If the behavior of applications, network protocols or operating systems is related with an interrupts-intensive activity, our technique can be much more suitable. Therefore, we claim our technique is a good candidate for the simulation of scalable networked event-driven systems.

In this paper, we validated our technique by implementing it with NQEM, a cycle-accurate discrete-event simulator. Experimental results show that our technique can improve simulation speed. We also compared our technique with AvroraZ [12]. The NQEM_Speedup is 2 to 11 times faster than AvroraZ. This paper is organized as follows: in Section 2, we describe the motivation of this study and how we reduce the overheads. The implementation details are explained in Section 3, and performance evaluations are discussed in Section 4. Related works are described in Section 5, and we present our conclusions in Section 6.

## 2. Enhancing the Speed of Simulation

### 2.1. Problem Definitions

Sensor network simulation requires high-fidelity and scalability. Previous studies on sensor network simulators have applied instruction-level and discrete-event simulation. Instruction-level simulation can maintain cycle-accurate simulation for high-fidelity and the discrete-event method can reduce the computation overheads during sleep states of virtual sensor nodes for scalability. However, they have same problems concerning the synchronization overhead between virtual sensor nodes and peripherals. Virtual sensor nodes have to be synchronized to ensure the causality when the nodes and peripherals communicate with each other. There are various synchronization methods to maintain the causality in the conservative approach such as the lock-step and shared queue techniques. However, according to our experimental observations, both these techniques also have synchronization overhead. In this section, we consider the problems in both techniques, and then our proposed method is explained.

#### 2.1.1. Lock-Step Technique

In the lock-step technique, since all of the simulated virtual sensor nodes have their own thread, every virtual sensor node has to wait for others to arrive at the lock-step point which is predetermined in time. However, that leads to a simulation with all of them delayed. This delay time and context-switching of every thread incur significant synchronization overheads. We identified the synchronization overhead between virtual sensor nodes by lock-step via experiments. Table 1 shows the average utilization of quad cores during the simulations of MoteWorks [13] CountSend/CountReceive applications. The configuration was 1-sender and N-receivers on AvroraZ. According to increase of virtual sensor nodes, the utilization of the processor cores is getting lower. The virtual sensor nodes spend most of their time waiting for other sensor nodes at lock-step points. That means the processors consume lots of time for context-switching rather than in computation. The synchronization overhead in this type of simulators occurs due to the lock-steps points. Therefore, by reducing these points, the simulation speed can be improved in this type of simulators.

#### 2.1.2. Shared Queue Technique

Classically, the shared queue approach is used for discrete-event simulation engines. When this approach is applied to network simulations, each node is identified using a shared event queue. Because every event generated during a simulation enters into the event queue according to its arrival time synchronized with other events, the event queue management incurs synchronization overhead. The simulation controller extracts events from the queue head since they are ordered by their arrival time. There are no delays, context-switching overheads or process blockings involved for the synchronization like in the lock-step technique. However, as the number of virtual sensor nodes is increased, the length of the event queue is also increased. As the result, the event queue management leads to larger synchronization overheads.

**Figure 1.**
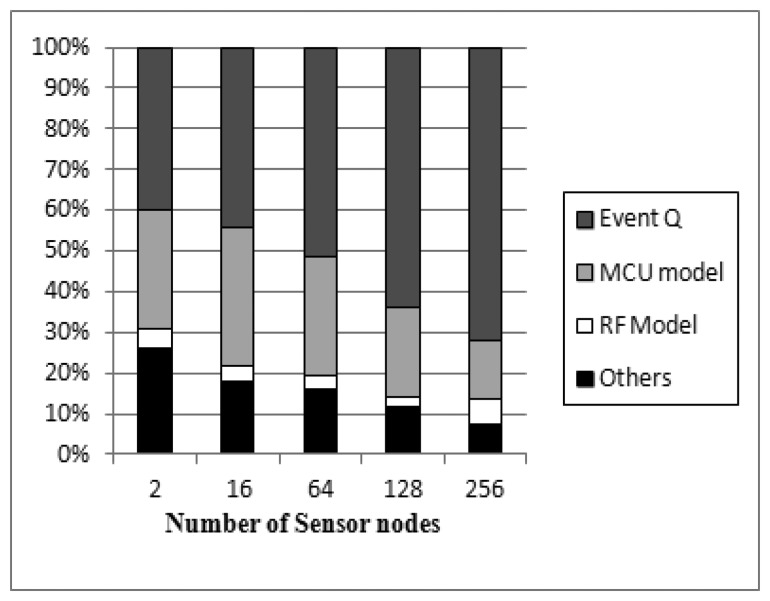
Profiling result of NQEM simulator.

Figure 1 shows the profiling result of simulation overheads in NQEM while simulating CntToRfm/RfmToLeds with 1-to-N configuration. The simulation engine of NQEM is SMPL [14] which is a discrete-event simulation language with the shared queue approach. We have measured the execution time of each function within the simulator. As NQEM is an instruction-level simulator, we expected the micro controller unit (MCU) model to take the largest computation resource. However, we found that the event queue management was the largest. The queue overhead increased as we increased the number of simulated nodes. The event queue management is the synchronization overhead in this case. Thus, the simulation speed can be enhanced by cutting the event queue consumption.

### 2.2. Optimistic Simulation of Interrupt Service Routines

The applications of networked sensors follow event-driven programming practices. Most of the time sensor nodes stay in the sleep state. When an event occurs by interrupts, they wake up. The interrupts are from its I/O devices such as a radio controller, sensors and actuators. The simulation of ISRs costs computation time for a large part of the code simulation in that environment.

Interrupt service routines usually execute time-critical codes. They are written efficiently to avoid potential preemptions by other interrupts. If no interrupts occur over a period of execution, the program execution sequence of each node is prefixed. However, each sensor node should communicate with sensors, actuators and a radio controller. Thus, most sensor network simulators exploit conservative approaches for their timing causality.

In this research, we propose an asynchronous simulation technique in order to reduce the synchronization overheads. The proposed technique is based on the optimistic simulation approach in ISRs instead of the conservative approach. The optimistic approach is not concerned with the causality between simulated events or sensor nodes. We assume the synchronization process for the sensor nodes is not necessary while an ISR of any sensor node is being simulated. If we can identify a code segment that is executed without any exceptional control flow, simulating the code can be asynchronous because the invoked events must be temporally executed. However, the exceptional control flows usually occur when sensor network applications are event-driven by communication controllers, sensors and actuators. According to experiments, we have observed only a few interrupt preemptions during execution of ISRs in our experiments. This means we have many opportunities to try the asynchronous simulation to avoid synchronizations between sensor nodes. Thus, we expect the ISRs within applications to be simulated asynchronously with only limited exceptions. This is the key point in improving simulation speed. However, when preemptions occur during this asynchronous simulation of ISRs, a causality error occurs and a recovery procedure is needed at that time.

**Figure 2.**
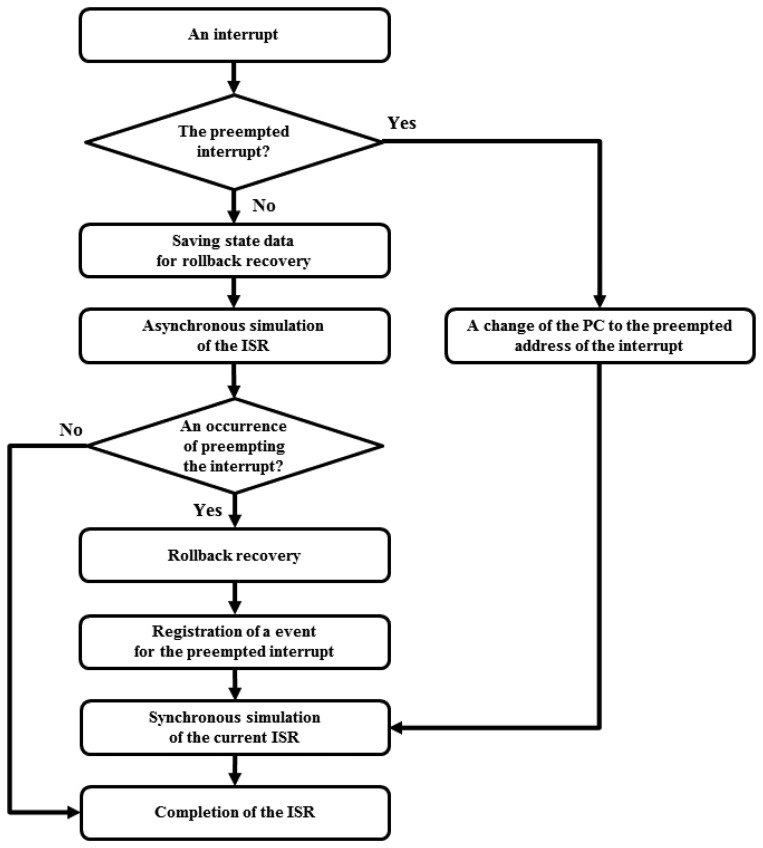
Asynchronous simulation of ISR.

Figure 2 shows the overall processing flow of the asynchronous simulation of an ISR. When the asynchronous simulation is started, the data structure for the virtual sensor should be saved. The data structure consists of virtual program counter, registers, flash memory, SRAM, external memory and simulation setting values. They are needed for the recovery procedure. Then the asynchronous simulation of the ISR for the sensor node is started. After the ISR is completed, the sensor node should wait for the carried timestamp of the simulation engine until the original synchronized simulation time reflecting the ISR's simulation time. If another interrupt which should have occurred during the ISR of the sensor node occurs before the simulation time, while the asynchronous ISR has been already completed, the simulator detects the causality error. At this point, the rollback recovery for the sensor node is performed. The saved data structure of the sensor node at the start of the ISR is restored, and then the sensor node is re-simulated to the ISR asynchronously until the point where the preemption should occur. Other synchronized sensor nodes can be simulated up to the point independently, and then the nodes wait for others. The preempting ISR is not emulated asynchronously to simplify the recovery procedure.

The causality error for a sensor node during asynchronous simulation of an ISR affects only its own simulated result and the synchronized simulation results of other sensor nodes are error free by the asynchronous simulation of the sensor node. In addition, because the virtual sensor nodes are modeled in the discrete-event simulation engine, the simulator can automatically detect causality errors between whole events from all the sensor nodes. Hence, each sensor node can perform the asynchronous simulation and the recovery mode independently and simultaneously. Our technique shows better performance where the ISR is long. As the length of ISR becomes longer, we can further avoid synchronizations between sensor nodes.

The proposed technique can be applied to any conservative simulation because our technique focuses on how to simulate interrupt service routine without the synchronization. This does not depend on any specific simulator architecture. For example, during simulation of ISRs, we remove the synchronization points in the lock-step technique or we do not assign any events into the event queue in the shared queue approach. Other simulators using any synchronization method also can apply this asynchronous simulation for ISRs. Therefore, this is an effective general approach for enhancing the sensor network simulation speed since there are a few preemptions between interrupts.

## 3. Implementation

In this section, we describe how we implemented the proposed technique. First, we explain our implementation of the optimistic simulation approach of ISR. Second, the rollback recovery procedure is explained.

### 3.1. Asynchronous Simulation of Interrupt Service Routines

**Figure 3.**
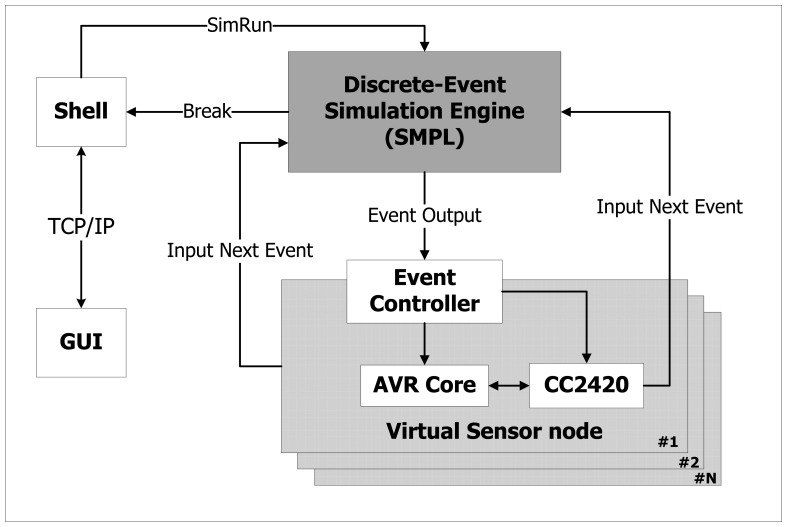
The architecture of a discrete-event simulator NQEM [6].

We have proved and validated our technique by implementing it with NQEM. It simulates a MicaZ mote which consists of an Atmega128 processor and CC2420. Figure 3 shows the NQEM architecture. The simulator uses SMPL as its discrete-event simulation engine which contains an event queue to maintain causality.

**Figure 4.**
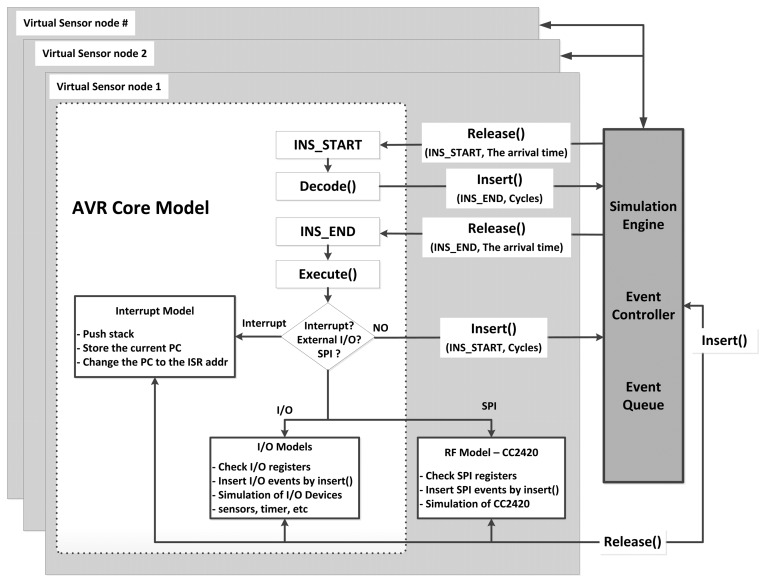
Synchronized simulations using the event queue on the SMPL engine.

Each virtual sensor node shares a single event queue for synchronized simulations, so instruction cycles are the base events to be processed by the queue. Events from different nodes are identified by SMPL's tokens. All virtual nodes generate events while they simulate the sensor nodes. Future events with their arrival times are inserted into the queue, and then the inserted events are sorted by their arrival times. The simulation clock proceeds whenever the simulator removes one event from the queue. After the removed event is processed, the results of the event are reflected into the virtual nodes. The simulator starts by inserting each sensor node's first instruction into the queue with different arrival times. Figure 4 shows the simulator's sequence to execute the base event as one instruction of a virtual node*. Insert()* and *Release()* are the operations to insert and retrieve the event from the queue. Figure 4 indicates that the procedures of modeled sensor nodes can be executed by *Insert()* and *Release()*. Every sensor node writes events to the queue and the simulation time progresses to the arrival time of the events as they are released. This sequence is repeated during the simulation.

**Figure 5.**
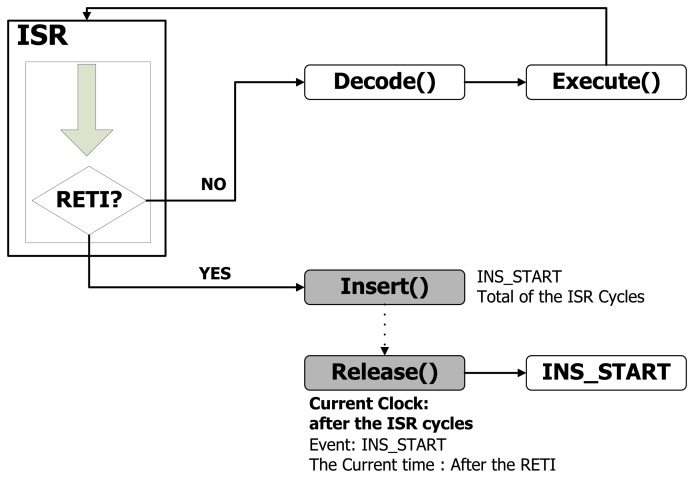
Optimistic asynchronous simulation of ISR without accessing the event queue.

Figure 5 shows the implemented optimistic simulation sequence. To simulate ISRs asynchronously, we should not access the event queue during the ISR period. While a regular instruction-level simulator repeats *Insert()* and *Release()* in an ISR, the implemented simulator simulates ISR instructions without calling *Insert()* and *Release()* which are the two functions for managing the event queue. The simulator executes ISR instructions without accessing the event queue until the *RETI* instruction is met. Through this technique, the simulator reduces the number of *Insert()* and *Release()* calls. As a result, event queue overhead for synchronization is improved. For the first instruction after the *RETI*, the simulator schedules that instruction event with its previous arrival time added by the total ISR's simulation time. The following instruction of the asynchronous ISR is likely to be scheduled at the end of the queue. This instruction event does not affect other sensor nodes running synchronously. This is reason why when an event is inserted into the event queue, the SMPL searches and verifies the event's causality before an insertion.

### 3.2. Rollback Recovery

**Figure 6.**
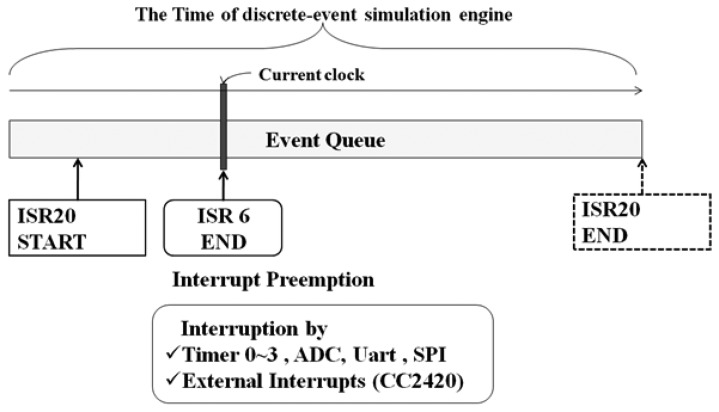
An interrupt preemption scenario in optimistic simulation.

Asynchronous simulation takes an optimistic approach for the ISRs assuming that there will be no preempting interrupts during the ISR period. However, when one or more preemptions occur, the simulator should detect it, and then a safe rollback process is performed. Figure 6 shows an interrupt preemption scenario. The simulator is processing *ISR20_START* event and performs asynchronous simulation as it is an ISR of the sensor node. *ISR20_END* event is inserted at the end of the queue reflecting ISR20's simulation time. At the position of *Current Clock*, another interrupt *ISR6* occurs and preempts *ISR20_START* while the asynchronous ISR has been already completed. The simulator detects the preemption by comparing the *Current Clock* and the simulation time of *ISR20_END*. If the *Current Clock* is earlier than the *ISR20_END*, the simulator determines that preemption should have occurred. Once the simulator knows that preemption has occurred, the rollback recovery routine is called. The routine restores the state data saved at the entry of the ISR, and the ISR is re-simulated up to the *Current Clock* position. Then, we can continue the synchronized simulation. After the rollback process, some events in the queue should be removed since they are inserted by the wrong optimistic simulation. Instead of explicitly removing them, we simply check the correctness of the events when they are released. This can reduce extra queue access overhead.

The rollback procedure consists of the rollback preparation and the rollback recovery. For the rollback preparation, each virtual sensor node needs to take some time and incurs in extra memory costs. The data structure for a virtual sensor node consists of virtual program counter, registers, flash memory, SRAM, external memory and simulation setting values. The extra memory cost is about 132 kB per one sensor node. As the number of virtual sensor nodes is increased, the memory overhead is increased. We have measured the rollback preparation time. The time consumption of saving the data structure is about 8 μs. Even though it is constant value, the overhead can affect to the speed up more as the length of an ISR is shorter. In the rollback recovery procedure, the simulator restores the saved data structure and removed events data for the sensor node, and then the sensor node is re-simulated. In addition, there is another side effect. Some virtual sensor nodes may take some extra time to wait for the sensor node during the recovery. The time for restoring data structure is the same with the preparation time as 8 μs. However, the number of re-simulating events and the extra time needed by the side effect are non-deterministic. They depend on the application's behavior and the simulation status of other virtual sensor nodes. Since the rollback recovery is called by the casualty errors, how many interrupt preemptions are invoked is important for the speedup.

## 4. Evaluation

We present the experiment-based evaluation of our proposed technique in this section. Three experiments were performed. First, we profiled interrupt service routines in sensor network applications to assess achievable speed improvements using our techniques. Second, we evaluated the speed up of our implementation. Third, we compared the scalability between AvroraZ and NQEM with the proposed technique.

All experiments were performed on an Intel i7 920 quad core desktop computer, running at 2.67 GHz clock with 4 GB memory. Profiling was performed on Windows XP platform. CentOS9 of Linux kernel 2.6.9 with JVM 1.6 was used to run AvroraZ. The six applications in Table 2 are selected for evaluation. We choose active applications in order to get the effective result in a short period of simulation time. All of the applications which are compiled MicaZ mote. CountSend, CountReceive from the MoteWorks 2.0 distribution and CntToRfm, CntToLedsAndRfm, SenseToRfm, RfmToLeds from the TinyOS 1.1.7 distribution. According to [15], MoteWorks distribution has various benefits over TinyOS 1.x or TinyOS 2.x.

### 4.1. Assessment of Achievable Speed up

The speed up is determined by the ISR length and number of invoked interrupts in our approach. Achievable enhancement can be estimated by profiling the amount of ISR instructions during the simulation time period. The profiling of each application was performed for the simulation time of 10 virtual seconds. Table 2 shows the results of each application for ISRs. CountSend and CountReceive of MoteWorks show an ISR ratio of 39% while TinyOS applications have the lower ISR ratio. Therefore, the simulation speed of MoteWorks applications can be faster than the TinyOS applications in our approach because of the ISR ratio.

We configured the networks as 1-sender/N-receivers and N-senders/N-Receivers using the applications. Experiments were performed with various numbers of nodes. The number of simulated instructions, number of ISR instructions, number of interrupts, and number of preemptions were recorded during the simulation. To count the number of instructions in ISR, we counted instructions within ISRs until the execution of the *RETI* instruction. The preemption count is increased when an interrupt is invoked before the execution of *RETI* instruction. The interrupt ratio was computed by dividing the number of ISR instructions by the number of simulated instructions.

Table 3 shows the profiling results with one sender and N receivers configuration. MoteWorks applications show an interrupt ratio of 39% and this value is almost constant over various numbers of nodes. However, for others, it also increases as the number of nodes increases. The reason is that the sender and the receiver nodes do not have the same ISR ratio. In Table 2, CountSend and CountReceive show the same ISR ratio of 39% while the receiver RfmToLeds has 12% and the senders CntToRfm, CntToLedsAndRfm and SenseToRfm have 3% to 5%. Hence, the ISR ratio of TinyOS applications increases as the number of nodes increases. Table 4 shows the results with N senders and N receivers configuration. Since the number of senders is increased, the number of invoked interrupts in the receivers is larger than that of 1-to-N configuration. The MoteWorks applications show a much larger increment in ISR ratio than the TinyOS applications because they have longer ISRs. According to Table 3 and Table 4, a few preemptions were recorded in this experiment. With SensToRfm(64 nodes)/RfmToLeds(64 nodes), only 93 preemptions occurred out of 296,931 interrupts. Thus, the rollback recovery routine was executed only a few times. The interrupt preemption count of SenseToRfm(64 nodes)/RfmToLeds(64 nodes) that has 11% ISR ratio is larger than CountSend(64 nodes)/CountReceive(64 nodes) that has a 60% ISR ratio. Though the ISR ratio is larger, the interrupt preemption count can be smaller. The number of preemption count is dependent on the application's architecture and behavior. According to the profiling results about the ISR ratio and the preemption count of the each application, we can estimate the speed up limit.

### 4.2. Evaluation of Improved Simulation Speed

**Figure 7.**
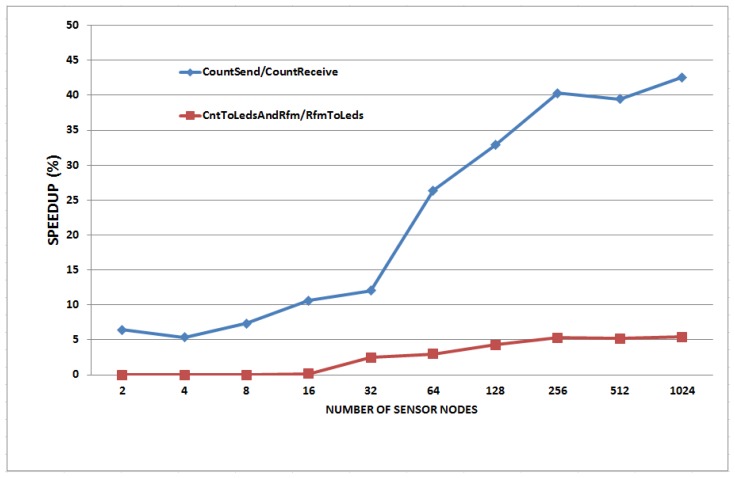
Simulation speedup with various numbers of nodes (1 sender, N receivers).

**Figure 8.**
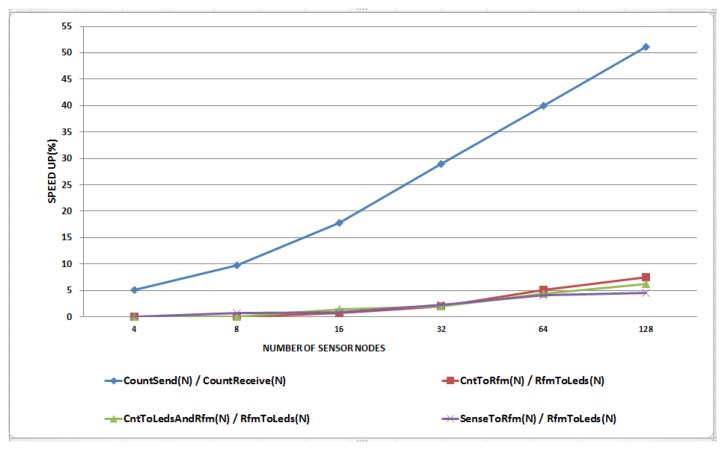
Simulation speedup with various numbers of nodes (N senders, N receivers).

We also evaluated the improvement of simulation speed in comparison with the basic NQEM. Simulations were performed for 10 virtual seconds of simulation time. Figure 7 and Figure 8 show the simulation speed up with various numbers of sensor nodes in 1-to-N and N-to-N configuration.

The Figure 7 shows two graphs. The upper plot is the result of MoteWorks applications and the bottom plot is from TinyOS applications. MoteWorks applications achieved more speed up than TinyOS applications and this is due to their large ISR ratio. In 1-to-N configuration, CountSend/CountReceive has an ISR ratio of 39% and CntToLedsAndRfm/RfmToLeds has an ISR ratio of 6% to 12% as shown in Table 3. This result shows that the monitored speed up values of the CountSend/CountReceive are quite close to the assessed achievable speed up values of almost the ISR ratio of 40% which is evaluated in Section 4.1. For the same reason, for the TinyOS applications, we had expected a 12% speed up with 12% of the ISR ratio. However, they achieved only a 6% speed up. This is because the rollback preparation overhead reduces the speed up gains. According to Table 3, each MoteWorks and TinyOS application performed 600 instructions and 75 instructions per one interrupt service routine, respectively. Though the numbers of interrupts invoked in those two test sets are similar and they have similar rollback preparation overheads, the speed up of TinyOS applications is further degraded due to the ISR ratio. In addition, the synchronization overhead increases exponentially, as the number of the sensor nodes increases according to our observations as shown in Table 1 and Figure 1.

**Figure 9.**
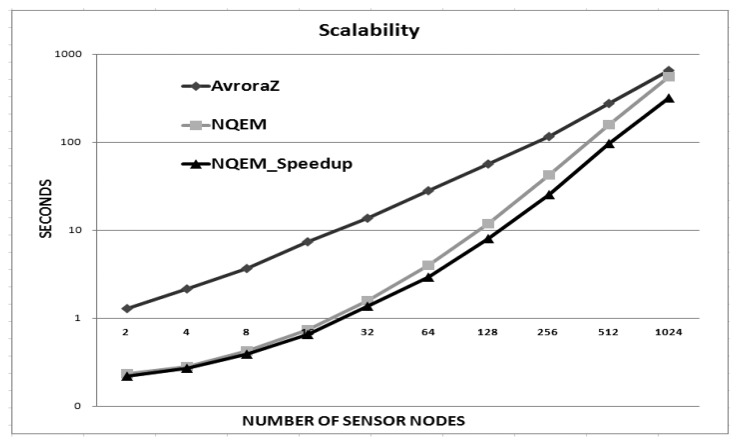
Scalability comparisons of NQEM and AvroraZ.

Avrora and NQEM show the same results by the synchronization issue even though they are implemented in the different techniques, since the graph represents that *y*-axis is log scale as the execution time in Figure 9. Figure 8 shows the speed up results in the N-to-N configuration. Three TinyOS application sets were simulated with the MoteWorks test set. More interrupts were invoked and a few preemptions are observed as the number of senders increased. Compared to the 1-to-N case, higher speed up was achieved. As Table 4 shows, the ISR ratio of CountSend/CountReceive is about 60% and we can see that the speed up approaches this limit. TinyOS applications show a smaller speed up as they have an ISR ratio ranging from 8% to 11%. For the same reasons we have mentioned concerning Figure 7, the expected speed up is not achieved. However, among the three TinyOS application sets, SenseToRfm/RfmToLeds shows the smallest speed up with 128 virtual nodes as shown in Table 4. This is due to the fact that it has the largest number of preemptions and their corresponding rollback recovery overheads. We showed that our method can enhance the simulation speed up. The speed up is determined by the behavior of applications, network protocols or operating systems. When they have long ISRs without preemptions between ISRs, our technique is more suitable.

### 4.3. Comparison with AvroraZ

We compared AvroraZ and NQEM. AvroraZ is an extension of Avrora with IEEE 802.15.4 support. We simulated the MoteWorks CountSend/CountReceive applications on AvroraZ, NQEM and NQEM_Speedup. The simulation was performed for 10 virtual seconds. The simulation result in 1-sender and N-receivers configuration of MicaZ motes is shown in Figure 9. The *y*-axis is the execution time and the *x*-axis is the number of virtual sensor nodes. As we show in this experiment, the optimistic simulation technique further improves the scalability, and our NQEM outperforms AvroraZ up to 1,024 nodes. The execution time of NQEM_Speedup was 0.65 s in 16 nodes and 318.79 s in 1,024 nodes while AvroraZ requires 7.32 s in 16 nodes and 655.11 s in 1,024 nodes. NQEM_Speedup is 11 times faster in 16 nodes and two times faster in 1,024 nodes than AvroraZ. Moreover, it can achieve real-time performance with 8 s in 128 sensor nodes. When we consider the fact that networked sensors use a limited number of communications to save lifetime, our approach can be a very helpful technique for simulating large scale sensor networks.

## 5. Related Works

The first tools used to simulate sensor networks are classical network simulators such as NS-2 and OMNET++. In recent years, underwater wireless sensor networks simulators such as Aqua-Sim [16], ns-miracle [17], and Climent [18] were presented. Aqua-Sim and ns-miracle are extended on NS-2 and Climent to provide a network simulation package based on NS-3 [19]. However, this type of simulators cannot simulate target applications. They can verify modeled protocols or behaviors so that actual applications cannot be applied.

TOSSIM [20] was developed as a simple TinyOS simulator. TOSSIM has an advantage in simulation speed over other simulators because it works with state transitions instead of working on the cycle-accurate instruction-level. Since it was designed only for TinyOS, it cannot support other operating systems. In addition, TOSSIM cannot simulate with fine-grain timing and code interrupts.

Cycle-accurate, instruction-level simulation is the known solution for high-fidelity. ATEMU [5] was the first instruction-level sensor network simulator. However, the simulator framework has poor scalability due to its fine-grain simulation process. NQEM [6], Avrora [7], Polarlite [10], SnapSim [11], *etc.*, are also instruction-level sensor network simulators, which can solve synchronization issues in various ways so that they can be faster than ATEMU.

NQEM is high-fidelity instruction-level simulator, and it can be extended to the power estimator [21]. It has greatly reduced the simulation overheads by using a discrete-event based SMPL simulation engine which contains an event queue to maintain conservative manner. However, the number of virtual sensor nodes are increased, the length of the event queue is increased for synchronization.

Avrora is well known instruction-level simulator. It uses the lock-step technique for synchronization between all virtual sensor nodes. However, large-scale context-switching can incur in overheads, because each virtual sensor node has its own thread.

Polarlite is an improved simulator which is based on Avrora. It introduced speedup techniques which use a longer synchronization interval than Avrora's constant interval strategy at the radio, MAC layer, and sleep mode. However, the synchronization strategy can be applied only if one of the scenarios in their study occurs and it still has large-scale context-switching overhead.

SnapSim is also based on Avrora. It uses an optimistic approach to reduce synchronizations about network transmission. However, It does not consider event-driven practices consisting of various networked sensors. Moreover, the method is dependent on the sensor network communication layers, so it cannot be extended to other simulation tools.

DiSenS [22] can be used for simulation not only for SMP, but also for cluster environments. The synchronization overhead is reduced by using network topology information. However, the communication overhead for managing synchronization can be a problem between clusters.

Cooja [23] provides both network level, operating system level and instruction level simulations. This feature has several advantages over traditional simulations restricted to one level for tests and developments. Furthermore, Cooja is extended for the interconnection of simulated sensor nodes and real node hardware [24]. Even though this tool offers efficient simulation environments, MSPSim [25] which is used as the instruction set simulator in the Cooja did not consider its speed up for the synchronization issue. In addition, it supports MSP430 microcontroller and our simulator has the Atemega 128 model, so the performance cannot be compared due to the different instruction-level simulations.

By comparison, the synchronization management is the most important technique for enhancing speed up. We reduce the overhead by exploiting asynchronous code segments during simulations of ISRs. This is an effective and general approach, and besides it can be extended to any instruction-level conservative simulation.

## 6. Conclusions and Future Works

In this paper, we have proposed a novel technique that improves simulation speed. It is based on an optimistic approach by asynchronously simulating ISR. A rollback procedure is implemented to restore the simulator back when it meets the timing causality error. We implemented the proposed technique on NQEM and reduced overall overhead. In the evaluation, our technique shows better performance when the ISR is long. We also compared our technique with the well-known sensor network simulator, and our method showed better scalability. This technique does not depend on any specific instruction-level conservative approach, so that it can be extended to other simulators. In the future works, we will apply our approach to other instruction-level simulators.

## Figures and Tables

**Table 1.** The average utilization of quad cores during AvororaZ simulation via lock-step technique for synchronization (CountSend / CountReceive, 1-to-N).

**# of Nodes**	**4**	**8**	**16**	**32**	**64**	**128**	**256**	**512**	**1,024**
Avg Utilization (%)	39	37	32	29	27	27	27	26	26

**Table 2.** Profiling results of sensor network applications for ISRs.

**Applications**	**# of Inst.**	**# of Inst. in ISR**	**ISR Ratio**	**# of Intr.**
MoteWorks 2.0				
CountSend	273,890	108,965	39.8 %	111
CountReceive	70,664	27,992	39.6 %	83
TinyOS 1.7				
CntToRfm	281,418	9,250	3.3 %	111
CntToLedsAndRfm	284,828	9,250	3.2 %	111
SenseToRfm	315,509	17,259	5.5 %	150
RfmToLeds	48,672	6,124	12.6 %	83

**Table 3.** Profiling results of sensor network applications for ISR (1 sender, N receivers).

**MoteWorks 2.0 - CountSend(1), CountReceive(N)**									
# of nodes	4	8	16	32	64	128	256	512	1,024
# of Instructions	487,154	774,366	1,347,754	2,494,375	4,789,981	9,369,175	18,517,175	36,839,580	73,492,899
# of ISR Inst.	193,256	306,274	532,100	983,752	1,887,581	3,692,614	7,299,950	14,520,817	28,964,441
Interrupt Ratio	39.7	39.6	39.5	39.4	39.4	39.4	39.4	39.4	39.4
# of Interrupts	363	705	1,387	2,751	5,484	10,925	21,781	43,552	87,112
# of Preemption	0	0	0	2	2	2	4	6	11
**TinyOS - CntToLedsAndRfm(1)/ RfmToLeds(N)**									

# of nodes	4	8	16	32	64	128	256	512	1024
# of Instructions	431,537	628,767	1,022,621	1,810,216	3,386,785	6,533,052	12,820,327	25,409,177	50,590,925
# of ISR Inst.	27,838	53,054	103,342	203,918	405,430	806,654	1,607,302	3,212,702	6,424,726
Interrupt Ratio	6.5	8.4	10.1	11.3	12.0	12.3	12.5	12.6	12.7
# of Interrupts	363	705	1387	2751	5484	10925	21782	43553	87112
# of Preemption	0	0	0	0	0	0	1	1	2

**Table 4.** Profiling results of sensor network applications for ISR (N senders, N receivers).

**MoteWorks 2.0-CountSend(N)/CountReceive(N)**								
# of nodes	16	32	64	128
# of Instructions	7,082,399	23,783,034	84,596,488	305,690,197
# of ISR Inst.	377,6879	13,559,491	50,255,546	185,836,411
Interrupt Ratio	53.3	57.0	59.4	60.8
# of Interrupts	5,833	21,256	79,492	295,307
# of Preemption	1	8	19	74
**TinyOS—CntToRfm(N)/RfmToLeds(N)**								

# of nodes	16	32	64	128
# of Instructions	5,628,263	1,774,8843	61,295,719	216,703,785
# of ISR Inst.	448,510	1,664,948	6,397,109	23,810,178
Interrupt Ratio	8.0	9.4	10.4	11.0
# of Interrupts	5,833	21,115	79,826	296,884
# of Preemption	0	0	15	41
**TinyOS—CntToLedsAndRfm(N)/RfmToLeds(N)**								

No of nodes	16	32	64	128
# of Instructions	5,656,607	17,845,480	61,409,073	21,715,0050
# of ISR Inst.	448,510	1,668,694	6,397,033	23,827,598
Interrupt Ratio	7.9	9.4	10.4	11.0
# of Interrupts	5,833	21,164	79,825	297,194
# of Preemption	0	1	12	34
**TinyOS—SenseToRfm/RfmToLeds(N)**								

# of nodes	16	32	64	128
# of Instructions	5,909,069	18,119,867	61,880,817	217,091,151
# of ISR Inst.	515,126	1,881,358	6,596,922	24,070,581
Interrupt Ratio	8.7	10.4	10.7	11.1
# of Interrupts	6,157	21,471	80,376	296,931
# of Preemption	0	7	30	93
